# Cyathane diterpenoids from fruiting bodies of *Phellodon niger*

**DOI:** 10.1007/s13659-011-0002-z

**Published:** 2011-09-05

**Authors:** Sheng-Tao Fang, Tao Feng, Ling Zhang, Ze-Jun Dong, Zheng-Hui Li, Ji-Kai Liu

**Affiliations:** 1 State Key Laboratory of Phytochemistry and Plant Resources in West China, Kunming Institute of Botany, Chinese Academy of Sciences, Kunming, 650201 Yunnan, China; 2 Graduate University of the Chinese Academy of Sciences, Beijing, 100049 China

**Keywords:** cyathane, diterpenoid, nigernin, *Phellodon niger*

## Abstract

Four new cyathane-type diterpenoids, nigernins C-F (**1–4**), together with four known compounds, were isolated from the fruiting bodies of the basidiomycete *Phellodon niger*. The structures of these new compounds were established on the basis of spectroscopic analysis, including 1D and 2D NMR experiments. In addition, nigernin F (**4**) with an unusual 3,4-*seco* cyathane diterpenoid skeleton was found to occur in nature for the first time. It was suggested to be as an oxidation product of C-3-C-4 bond cleavage of nigernin E (**3**).

**Electronic Supplementary Material:**

Supplementary material is available for this article at 10.1007/s13659-011-0002-z and is accessible for authorized users.

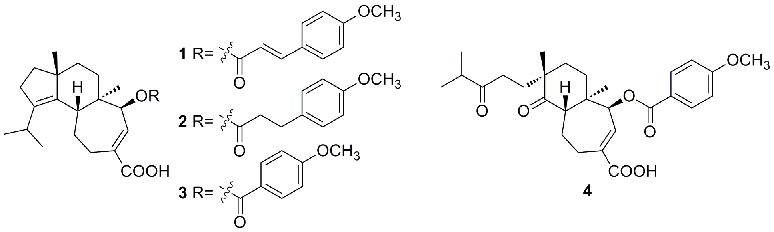

## Introduction

*Phellodon niger* is an edible fungus belonging to the family Hydnaceae.[1] In our continuing search for novel and secondary metabolites from higher fungi of Yunnan province in China, we have previously isolated two new cyathane diterpenoids, nigernins A and B from this fungus.[2] Further research for the cyathane-type diterpenoids in the fruiting bodies of *P. niger* led to the isolation of four new cyathanes, nigernins C–F (**1**–**4**), along with four known compounds, sarcodonin δ,[3] 1,2-diacetoxy-3-(4′-hydroxyphenyl)-4,7,8-trihydroxy-dibenzofuran (Bl-V),[4] grifolic acid[5] and uridine.[6] Herein, we report the isolation and structure elucidation of the new compounds.

## Results and Discussion

Compound **1** was isolated as white amorphous powder. The molecular formula was established to be $C_{30}H_{38}O_5$ based on HREIMS at *m/z* 478.2701 $[M]^+$ (calcd for $C_{30}H_{38}O_5$ $[M]^+$, 478.2719), indicating twelve degrees of unsaturation. The IR spectrum showed the presence of a hydroxy (3423 $cm^{-1}$) group, a benzene ring (1604, 1513, 1460 $cm^{-1}$) and two carbonyl (1713, 1690 $cm^{-1}$) groups. The $^1H$ NMR spectrum (Table 1) indicated the presence of four methyls [$\delta_H$ 0.88 (3H, s, H-16); 0.96 (6H, d, $J$ = 6.7 Hz, H-19 and 20); 1.12 (3H, s, H-17)], a methoxyl group at $\delta_H$ 3.84 (3H, s, 4′-$OCH_3$), a 1′,4′-disubstituted benzene ring [$\delta_H$ 6.91 (2H, d, $J$ = 8.7 Hz, H-3′ and 5′) and 7.49 (2H, d, $J$ = 8.7 Hz, H-2′ and 6′)], a trans-double bond [$\delta_H$ 6.34 (1H, d, $J$ = 15.9 Hz, H-8′) and 7.69 (1H, d, $J$ = 15.9 Hz, H-7′)], an olefinic proton at $\delta_H$ 7.22 (1H, d, $J$ = 7.1 Hz, H-13), and an oxymethine at $\delta_H$ 5.02 (1H, d, $J$ = 7.1 Hz, H-14). The $^{13}C$ NMR spectrum of **1** (Table 1) revealed the presence of one *p*-methoxycinnamoyloxyl moiety [$\delta_C$ 55.4 (q),

**4**

114.3 (d × 2), 115.1 (d), 127.0 (s), 129.8 (d × 2), 145.0 (d), 161.5 (s), 166.5 (s)]. The remaining 20 carbons were ascribable for four methyls, six methylenes, four methines, five quaternary carbons, and one carbonyl group. Comparison of NMR data of **1** with those for nigernin A, previously isolated from this fungus,[2] revealed the presence of the characteristic signals of a cyathane-type diterpenoid. The absence of a methylene resonance at $\delta_C$ 43.4 in the $^{13}C$ NMR spectrum of nigernin A, and the appearance of the signals at $\delta_C$ 77.8 and $\delta_H$ 5.02 (d, $J$ = 7.1 Hz) in the NMR spectra of **1**, suggested the existence of an oxygenated methine attributable to C-14 in **1**. This was supported by the coupling constant ($J$ = 7.1 Hz) of H-13 at $\delta_H$ 7.22, and the HMBC correlations from H-14 to C-5, C-12 and C-13, and from H-16 to C-14 (Figure

*To whom correspondence should be addressed. E-mail: jkliu@mail.kib.ac.cn

1). In addition, the HMBC correlation from H-14 to C-9′ suggested that the *p*-methoxycinnamoyloxyl ester unit was linked to C-14. The ROESY correlations between H-5 and H-17, H-17 and H-8$\beta$, H-16 and H-8$\alpha$, H-16 and H-14 were observed in **1**. It indicated H-14 to be $\alpha$-oriented (Figure 2).

**Figure 1.** Key $^1$H,$^1$H-COSY and HMBC correlations of **1** and **4**.

Thus, the structure of **1** was elucidated as 14$\beta$-(*p*-methoxycinnamoyloxyl)-cyatha-3,12-diene-15-oic acid, and named as nigernin C.

Compound **2** was obtained as white amorphous powder, giving the molecular formula $C_{30}H_{40}O_5$ by the HREIMS at *m/z* 480.2880 $[M]^+$ (calcd for $C_{30}H_{40}O_5$, $[M]^+$, 480.2876). The NMR spectral data of **2** (Table 1) were very similar to those of **1**, suggesting that **2** was also a cyathane diterpenoid. The key difference was the double bond in *p*-methoxycinnamoyloxyl unit of **1** replaced by two methylenes in **2**. This was confirmed by the HMBC spectrum, which showed correlations of H-7′ with C-1′, C-2′, C-6′, C-8′ and C-9′, and of H-8′ with C-1′, C-7′ and C-9′. In addition, correlation from $\delta_H$ 4.86 (1H, d, $J$ = 7.2 Hz, H-14) to $\delta_C$ 172.3 (s, C-9′) was also observed in the HMBC spectra, indicating that the 3-(4-methoxyphenyl)propanoyloxyl ester unit was also linked to C-14 of the cyathane skeleton. The stereochemistry of **2** was in accordance with **1** by the analysis of the ROESY spectrum. Consequently, the structure of **2** was determined as 14$\beta$-(3-(4-methoxyphenyl)propanoyloxyl)-cyatha-3,12-diene-15-oic acid, and named as nigernin D.

Compound **3** was isolated as white amorphous powder. Its molecular formula was determined to be $C_{28}H_{36}O_5$ on the basis of molecular ion peak at *m/z* 452.2554 in the HREIMS (calcd for $C_{28}H_{36}O_5$ $[M]^+$, 452.2563), in combination with the $^{13}$C NMR and DEPT spectra. The $^1$H and $^{13}$C NMR spectroscopic data of **3** (Table 1) were very similar to those of **1**, except for a *p*-methoxybenzoyloxyl group in **3** instead of the *p*-methoxycinnamoyloxyl group in **1**. The proton signals at $\delta_H$ 6.94 (2H, d, $J$ = 8.9 Hz, H-3′ and 5′) and 8.04 (2H, d, $J$ = 8.9 Hz, H-2′ and 6′) in the $^1$H NMR spectrum, together with the $^{13}$C-NMR signals at $\delta_C$ 55.5 (q), 113.8 (d × 2), 122.4 (s), 131.7 (d × 2), 163.5 (s), 165.5 (s) were determined readily as a *p*-methoxybenzoyloxyl unit. The location of the substituent and the stereochemistry of **3** were the same as those in **1** on the basis of analysis of HMBC and ROESY data. Therefore, compound **3** was identified as 14$\beta$-(*p*-methoxybenzoyloxyl)-cyatha-3,12-diene-15-oic acid, and named as nigernin E.

Compound **4** was obtained as white, amorphous powder and assigned the molecular formula $C_{28}H_{36}O_7$ as deduced by HREIMS (found *m/z* 484.2450 $[M]^+$, calcd for $C_{28}H_{36}O_7$ $[M]^+$, 484.2461). Comparison of the $^1$H and $^{13}$C NMR data of **4** (Table 1) with those of **3** indicated that their structures were similar. The main differences between these two compounds were the appearance of two keto carbonyl signals at $\delta_C$ 215.0 (s, C-3) and 215.5 (s, C-4) in **4** and the absence of two olefinic quaternary carbons [$\delta_C$ 140.4 (s, C-3) and 138.1(s, C-4)] in **3**. In addition, the EIMS of **4** with molecular ion peak $[M]^+$ at *m/z* 484 suggested more 32 mass units than that of **3**. On the basis of above evidence and the literature,[7] compound **4** should be a

**Figure 2.** Key ROESY correlations of compounds **1** and **4**.

3,4-*seco* cyathane diterpenoid due to an oxidation cleavage of C-3-C-4 double bond of **3**. This was also confirmed by HMBC correlations (Figure 1) from H-2, H-18, H-19 and H-20 to C-3, and from H-5 and H-17 to C-4. The ROESY (Figure 2) correlations between H-5 and H-17 of **4** indicated that the methyl at C-9 is $\beta$. Therefore, the structure of **4** was determined to be 3,4-*seco* nigernin E, and named as nigernin F. To the best of our knowledge, this is the first report of the 3,4-*seco* cyathane skeleton from nature.

## Experimental Section

**General Experimental Procedures.** Optical rotations were measured with a Horiba SEPA-300 polarimeter. UV spectra were recorded on a Shimadzu UV-2401PC spectrophotometer. IR spectra were obtained on a Bruker Tensor 27 spectrometer with KBr pellets. NMR spectra were recorded on a Bruker AV-400 or a DRX-500 spectrometer with TMS as an internal standard. EIMS and HREIMS were recorded on a VG Autospec-3000 mass spectrometer. Silica gel (200-300 mesh, Qingdao Marine Chemical Inc., China) and Sephadex LH-20 (Amersham Biosciences, Sweden) were used for column chromatography. HPLC was performed on an Agilent 1100 liquid chromatography system equipped with a Zorbax SB-$C_{18}$ column (9.4 mm × 150 mm). TLC was performed on silica gel plates (GF254, Qingdao Marine Chemical Inc., China). The spots on TLC were visualized by UV light (254/365 nm) and sprayed with 10% $H_2SO_4$ in ethanol, followed by heating.

**Table 1. $^1$H and $^{13}$C NMR (400/100MHz) data of 1–4 in $CDCl_3$ ($\delta$ in ppm, *J* in Hz).**

| Pos. | 1 | | 2 | | 3 | | 4 | |
| --- | --- | --- | --- | --- | --- | --- | --- | --- |
| | $\delta_H$ | $\delta_C$ | $\delta_H$ | $\delta_C$ | $\delta_H$ | $\delta_C$ | $\delta_H$ | $\delta_C$ |
| 1 | 1.59, m; 1.51, m | 37.8, $CH_2$ | 1.56, m; 1.47, m[b] | 37.7, $CH_2$ | 1.59, m; 1.50, m | 37.8, $CH_2$ | 1.70, t (7.4) | 32.2, $CH_2$ |
| 2 | 2.29, t (7,5) | 28.5, $CH_2$ | 2.26, t (7.5) | 28.5, $CH_2$ | 2.29 (t, 7.6) | 28.6, $CH_2$ | 2.53, m; 2.45, m | 35.4, $CH_2$ |
| 3 | | 140.3, qC | | 140.3, qC | | 140.5, qC | | 215.0, qC |
| 4 | | 138.2, qC | | 138.1, qC | | 138.1, qC | | 215.5, qC |
| 5 | 3.06, m | 44.0, CH | 2.91, m | 43.8, CH | 3.17, m | 44.2, CH | 3.46, m | 51.6, CH |
| 6 | | 41.5, qC | | 41.2, qC | | 41.6, qC | | 43.9, qC |
| 7 | 1.99, m | 33.9, $CH_2$ | 1.77, td (13.4, 4.3) | 33.8, $CH_2$ | 2.04, m | 34.1, $CH_2$ | 2.38, m | 32.2, $CH_2$ |
| | 1.18 br, d (13.5) | | 1.04, m | | 1.19 br, d (13.8) | | 1.37, d (13.5) | |
| 8 | 1.55, m | 36.5, $CH_2$ | 1.47, m[b] | 36.4, $CH_2$ | 1.54, m | 36.5, $CH_2$ | 1.78, m | 33.1, $CH_2$ |
| | 1.42 br, d (13.5) | | 1.34 br, d (11.8) | | 1.40 br, d (12.5) | | 1.59, m | |
| 9 | | 49.2, qC | | 49.1, qC | | 49.2, qC | | 46.2, qC |
| 10 | 1.98, m | 26.2, $CH_2$ | 1.90, m | 26.2, $CH_2$ | 2.00, m | 26.4, $CH_2$ | 1.94 br, d (13.7) | 21.0, $CH_2$ |
| | | | | | | | 1.50, m | |
| 11 | 2.81, dt (15.5, 4.5) | 25.7, $CH_2$ | 2.72 br, d (15.4) | 25.6, $CH_2$ | 2.82 br, d (15.9) | 25.8, $CH_2$ | 2.91, dd (15.9, 4.5) | 24.2, $CH_2$ |
| | 2.57, m | | 2.34, m | | 2.56, m | | 2.39, m | |
| 12 | | 136.6, qC | | 136.8, qC | | 136.9, qC | | 137.7, qC |
| 13 | 7.22, d (7.1) | 141.6, CH | 7.10, d (7.2)[c] | 140.9, CH | 7.25, d (7.3) | 141.4, CH | 7.22, d (7.3) | 139.8, CH |
| 14 | 5.02, d (7.1) | 77.8, CH | 4.86, d (7.2) | 78.0, CH | 5.09, d (7.3) | 78.0, CH | 5.15, d (7.3) | 76.5, qC |
| 15 | | 172.5, qC | | 171.5, qC | | 172.3, qC | | 171.3, qC |
| 16 | 0.88, s | 16.5, $CH_3$ | 0.80, s | 16.4, $CH_3$ | 0.90, s | 16.5, $CH_3$ | 0.83, s | 16.8, $CH_3$ |
| 17 | 1.12, s | 24.3, $CH_3$ | 1.03, s | 24.2, $CH_3$ | 1.11, s | 24.3, $CH_3$ | 1.23, s | 23.6, $CH_3$ |
| 18 | 3.00, m | 26.8, CH | 2.95, m | 26.8, CH | 3.01, m | 26.8, CH | 2.63, m | 40.9, CH |
| 19 | 0.96, d (6.7) | 21.9, $CH_3$[a] | 0.93, d (6.7) | 21.9, $CH_3$[a] | 0.96, d (6.7) | 21.9, $CH_3$[a] | 1.09, d (6.9) | 18.3, $CH_3$ |
| 20 | 0.96, d (6.7) | 21.7, $CH_3$[a] | 0.93, d (6.7) | 21.6, $CH_3$[a] | 0.96, d (6.7) | 21.7, $CH_3$[a] | 1.09, d (6.9) | 18.3, $CH_3$ |
| 1′ | | 127.0, qC | | 132.2, qC | | 122.4, qC | | 121.9, qC |
| 2′ | 7.49, d (8.7) | 129.8, CH | 7.10, d (6.7)[b] | 129.2, CH | 8.04, d (8.9) | 131.7, CH | 8.01, d (8.5) | 131.6, CH |
| 3′ | 6.91, d (8.7) | 114.3, CH | 6.81, d (6.7) | 113.9, CH | 6.94, d (8.9) | 113.8, CH | 6.95, d (8.5) | 114.0, CH |
| 4′ | | 161.5, qC | | 158.1, qC | | 163.5, qC | | 163.7, qC |
| 5′ | 6.91, d (8.7) | 114.3, CH | 6.81, d (6.7) | 113.9, CH | 6.94, d (8.9) | 113.8, CH | 6.95, d (8.5) | 114.0, CH |
| 6′ | 7.49, d (8.7) | 129.8, CH | 7.10, d (6.7)[b] | 129.2, CH | 8.04, d (8.9) | 131.7, CH | 8.01, d (8.5) | 131.6, CH |
| 7′ | 7.69, d (15.9) | 145.0, CH | 2.93, t (7.6) | 30.3, $CH_2$ | | 165.5, qC | | 165.2, qC |
| 8′ | 6.34, d (15.9) | 115.1, CH | 2.66, t (7.6) | 36.2, $CH_2$ | | | | |
| 9′ | | 166.5, qC | | 172.3, qC | | | | |
| $OCH_3$ | 3.84, s | 55.4, $CH_3$ | 3.78, s | 55.2, $CH_3$ | 3.86, s | 55.5, $CH_3$ | 3.87, s | 55.5, $CH_3$ |

[a]Interchangeable assignments. [b]Overlapping resonances.

**Fungal Material.** The basidiomycete *P. niger* was collected at Wuding of Yunnan Province in August 2009, and identified by Prof. Zhu-Liang Yang, Kunming Institute of Botany. The voucher specimen was deposited at the Herbarium of Kunming Institute of Botany, Chinese Academy of Sciences.

**Extraction and Isolation.** The air-dried fruiting bodies (950 g) were extracted three times with $CHCl_3$/MeOH (1:1, v/v) at room temperature. After removal of the solvent by evaporation, the residue (98.0 g) was subjected to silica gel column eluted with a petroleum ether-acetone gradient system (1:0–1:1, v/v) to give fractions A–I. Fraction E was subjected to Sephadex LH-20 using $CHCl_3$-MeOH (1:1, v/v) to give 4 subfractions: E1–E4. Subfraction E3 was further purified by silica gel chromatography ($CHCl_3$/MeOH, 50:1) and preparative HPLC ($CH_3CN/H_2O$, 65:35) to obtain **2** (9.6 mg) and **3** (18.3 mg). Compound **4** (13.2 mg) was purified from subfraction E2 by preparative HPLC ($CH_3CN/H_2O$, 40:60). Fraction F was passed through Sephadex LH-20 using $CHCl_3$/MeOH (1:1, v/v) and repeated column chromatography over silica gel, and finally purified by preparative HPLC using a mobile phase of $CH_3CN/H_2O$ (75:25 and 65:35) to afford **1** (16.2 mg) and grifolic acid (12.3 mg), respectively. Sarcodonin δ (17.0 mg) was purified from fraction G by repeated silica gel column chromatography. Fraction H was subjected to silica gel, Sephadex LH-20, and preparative HPLC to give 1,2-diacetoxy-3-(4′-hydroxyphenyl)-4,7,8-trihydroxy-dibenzofuran (5.4 mg). Fraction I was separated over silica gel eluted with $CHCl_3$/MeOH (10:1), and then further purified by preparative HPLC to afford uridine (2.6 mg).

**Nigernin C (1):** white amorphous powder; $[\alpha]_D^{20} - 26.7$ (*c* 0.27, MeOH); UV (MeOH) $\lambda_{max}$ (log $\varepsilon$): 311 (4.09), 223 (3.97) nm; IR (KBr) $\nu_{max}$: 3423, 2958, 2935, 2866, 1713, 1690, 1604, 1513, 1460, 1252, 1170, 999, 828 $cm^{-1}$; $^1H$ and $^{13}C$ NMR data, see Table 1; EIMS *m/z* 478 $[M]^+$; HREIMS *m/z* 478.2701 $[M]^+$ (calcd for $C_{30}H_{38}O_5$ $[M]^+$, 478.2719).

**Nigernin D (2):** white amorphous powder; $[\alpha]_D^{20} - 2.64$ (*c* 0.24, MeOH); UV (MeOH) $\lambda_{max}$ (log $\varepsilon$): 223 (3.85) nm; IR (KBr) $\nu_{max}$: 3432, 2955, 2934, 2866, 1690, 1613, 1514, 1461, 1248 $cm^{-1}$; $^1H$ and $^{13}C$ NMR data, see Table 1; EIMS *m/z* 480 $[M]^+$; HREIMS *m/z* 480.2880 $[M]^+$ (calcd for $C_{30}H_{40}O_5$ $[M]^+$, 480.2876).

**Nigernin E (3):** white amorphous powder; $[\alpha]_D^{20} - 37.0$ (*c* 0.21, MeOH); UV (MeOH) $\lambda_{max}$ (log $\varepsilon$): 258 (3.94) nm; IR (KBr) $\nu_{max}$: 3424, 2958, 2936, 2866, 1717, 1690, 1607, 1511, 1459, 1257, 1167, 1098 $cm^{-1}$; $^1H$ and $^{13}C$ NMR data, see Table 1; EIMS *m/z* 452 $[M]^+$; HREIMS *m/z* 452.2554 $[M]^+$ (calcd for $C_{28}H_{36}O_5$ $[M]^+$, 452.2563).

**Nigernin F (4):** white amorphous powder; $[\alpha]_D^{20} + 33.4$ (*c* 0.21, MeOH); UV (MeOH) $\lambda_{max}$ (log $\varepsilon$): 259 (4.01) nm; IR (KBr) $\nu_{max}$: 3434, 2968, 2936, 2871, 1710, 1606, 1512, 1462, 1258, 1167, 1098 $cm^{-1}$; $^1H$ and $^{13}C$ NMR data, see Table 1; EIMS *m/z* 484 $[M]^+$; HREIMS *m/z* 484.2450 $[M]^+$ (calcd for $C_{28}H_{36}O_7$ $[M]^+$, 484.2461).

**Cytotoxicity Assay.** The cytotoxicity assay against C8166 cells ($CC_{50}$) was assessed using the MTT method and anti-HIV-1 activity was evaluated by the inhibition assay for the cytopathic effects of HIV-1 ($EC_{50}$).[19]

## Electronic Supplementary Material

Supplementary material is available in the online version of this article at http://dx.doi.org/10.1007/s13659-011-0002-z and is accessible for authorized users.

## Acknowledgments

This project was supported by the National Basic Research Program of China (973 Program, 2009CB522300), the National Natural Sciences Foundation of China (30830113), and MOST (2009ZX09501-029).

**Open Access** This article is distributed under the terms of the Creative Commons Attribution License which permits any use, distribution, and reproduction in any medium, provided the original author(s) and source are credited.

## Electronic supplementary material

Supplementary material, approximately 2.87 MB.
