# Supplementary material for: Cyathane diterpenoids from fruiting bodies of Phellodon niger
Source: Nat Prod Bioprospect. 2011 Sep 5;1(1):37–40. doi: 10.1007/s13659-011-0002-z (PMC4131705; doi:10.1007/s13659-011-0002-z)
Supplement: Supplementary file 1 — Supplementary material, approximately 2.87 MB. [file 13659_2011_2_MOESM1_ESM.pdf]

Electronic Supplementary Material

Cyathane diterpenoids from fruiting bodies of *Phellodon niger*

Sheng-Tao FANG,[a,b] Tao FENG,[a] Ling ZHANG,[a,b] Ze-Jun DONG,[a] Zheng-Hui LI,[a] and Ji-Kai LIU[a,]*

[a]State Key Laboratory of Phytochemistry and Plant Resources in West China, Kunming Institute of Botany, Chinese Academy of Sciences, Kunming 650201, Yunnan, China

[b]Graduate University of the Chinese Academy of Sciences, Beijing 100049, China

Received 14 June 2011; Accepted 24 July 2011

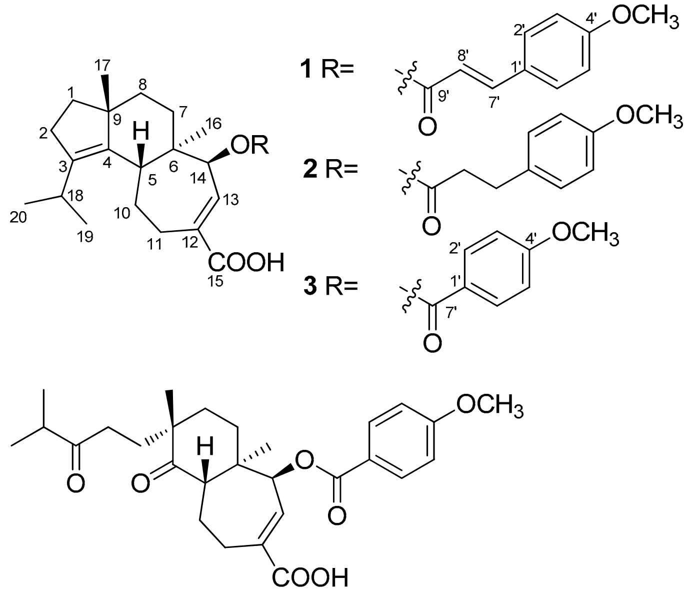

4

Structures of compounds **1**–**4**.

*To whom correspondence should be addressed. E-mail: jkliu@mail.kib.ac.cn.

Figure S1.1. ^{1}H NMR spectrum of compound **1** in CDCl3.

Figure S1.2. ^{13}C NMR spectrum of compound **1** in CDCl3.

Figure S1.3. HR-EI-MS of compound **1**.

Figure S1.4. ^{1}H,^{1}H-COSY spectrum of compound **1** in CDCl3.

Figure S1.5. HSQC spectrum of compound **1** in CDCl3.

Figure S1.6. HMBC spectrum of compound **1** in CDCl3.

Figure S1.7. ROESY spectrum of compound **1** in CDCl3.

Figure S2.1. ^{1}H NMR spectrum of compound **2** in CDCl3.

Figure S2.2. ^{13}C NMR spectrum of compound **2** in CDCl3.

Figure S2.3. HR-EI-MS of compound **2**.

Figure S2.4. ^{1}H,^{1}H-COSY spectrum of compound **2** in CDCl3.

Figure S2.5. HSQC spectrum of compound **2** in CDCl3.

Figure S2.6. HMBC spectrum of compound **2** in CDCl3.

Figure S2.7. ROESY spectrum of compound **2** in CDCl3.

Figure S3.1. ^{1}H NMR spectrum of compound **3** in CDCl3.

Figure S3.2. ^{13}C NMR spectrum of compound **3** in CDCl3.

Figure S3.3. HR-EI-MS of compound **3**.

Figure S3.4. ^{1}H,^{1}H-COSY spectrum of compound **3** in CDCl3.

Figure S3.5. HSQC spectrum of compound **3** in CDCl3.

Figure S3.6. HMBC spectrum of compound **3** in CDCl3.

Figure S3.7. ROESY spectrum of compound **3** in CDCl3.

Figure S4.1. ^{1}H NMR spectrum of compound **4** in CDCl3.

Figure S4.2. ^{13}C NMR spectrum of compound **4** in CDCl3.

Figure S4.3. HR-EI-MS of compound **4**.

Figure S4.4. ^{1}H,^{1}H-COSY spectrum of compound **4** in CDCl3.

Figure S4.5. HSQC spectrum of compound **4** in CDCl3.

Figure S4.6. HMBC spectrum of compound **4** in CDCl3.

Figure S4.7. ROESY spectrum of compound **4** in CDCl3.

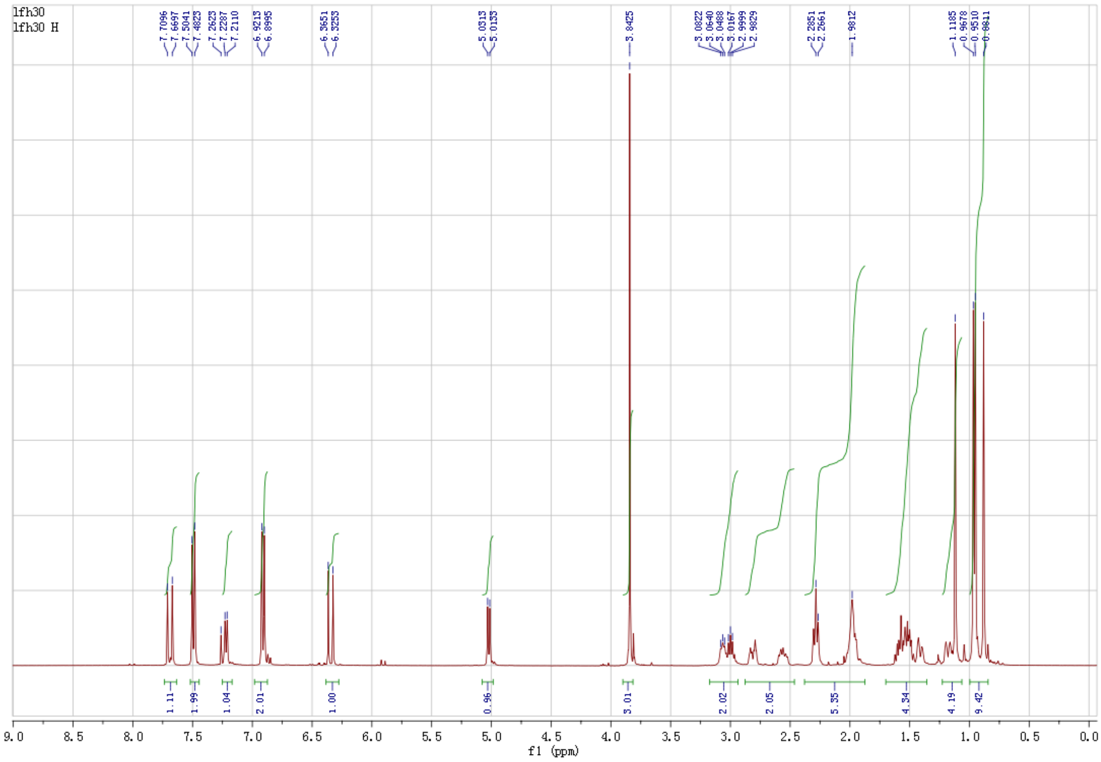

Figure S1.1. ^{1}H NMR spectrum of compound **1** in CDCl3.

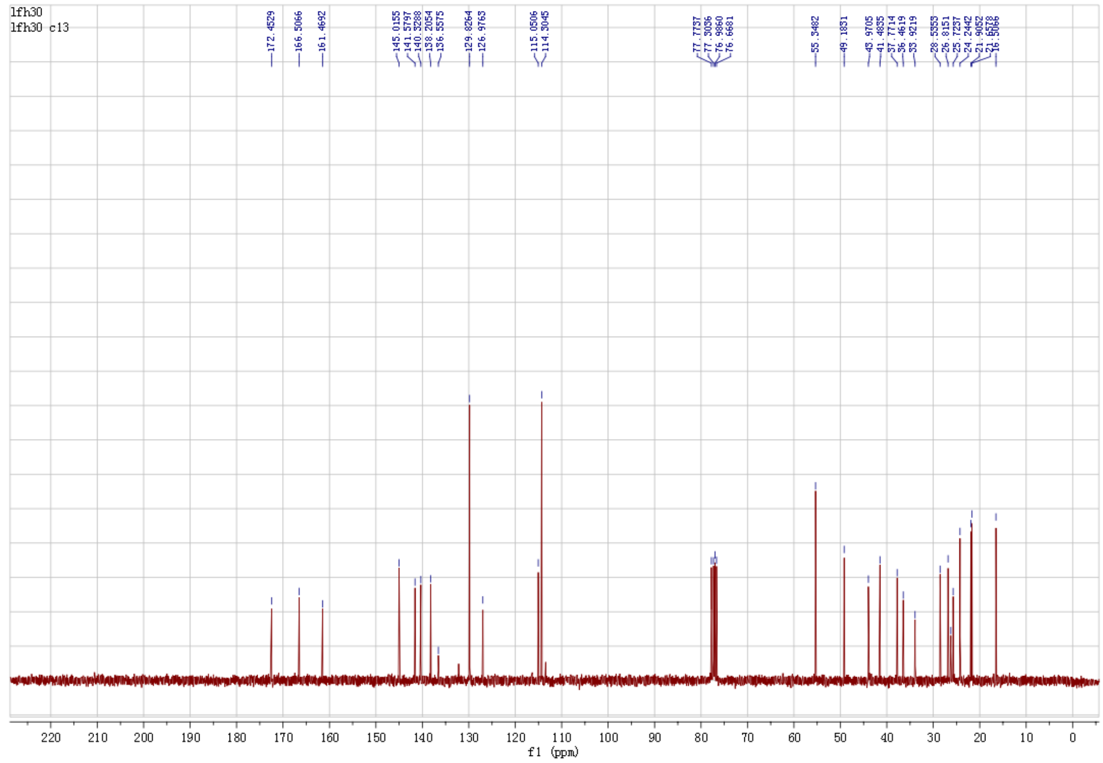

Figure S1.2. ^{13}C NMR spectrum of compound **1** in CDCl3.

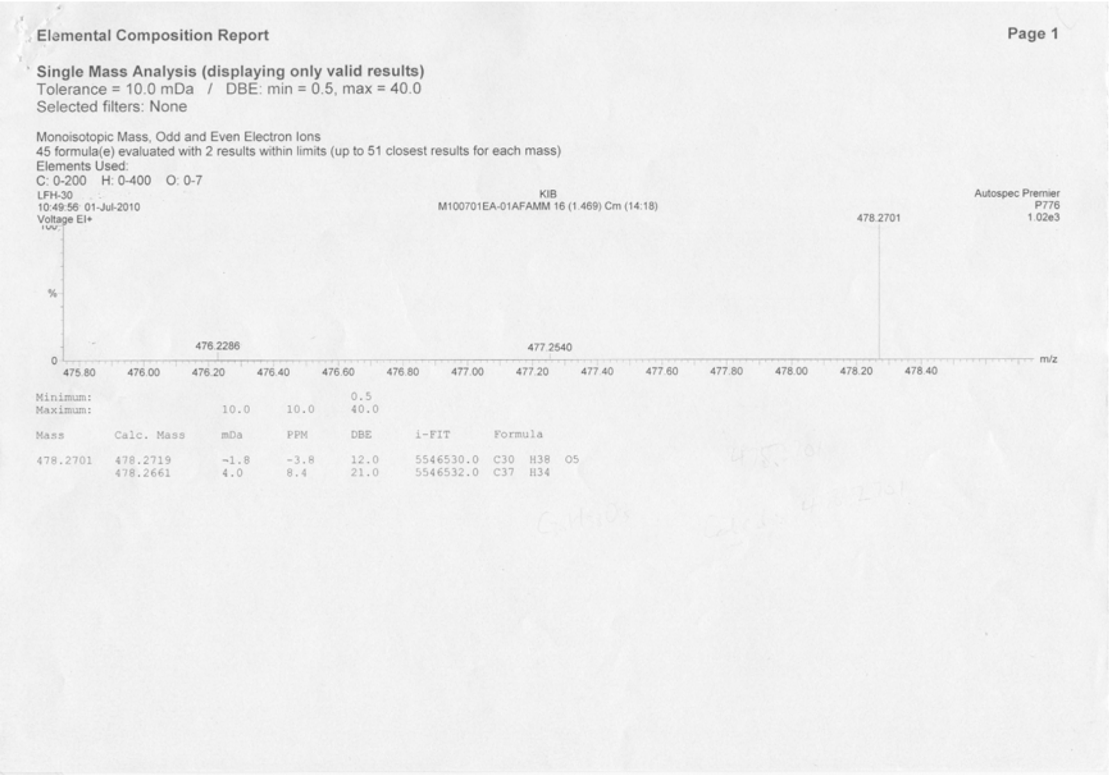

Mass	Calc. Mass	mDa	PPM	DBE	i-FIT	Formula
Minimum:				0.5		
Maximum:		10.0	10.0	40.0		
478.2701	478.2719	-1.8	-3.8	12.0	5546530.0	C30 H38 O5
	478.2661	4.0	8.4	21.0	5546532.0	C37 H34

Figure S1.3. HR-EI-MS of compound **1**.

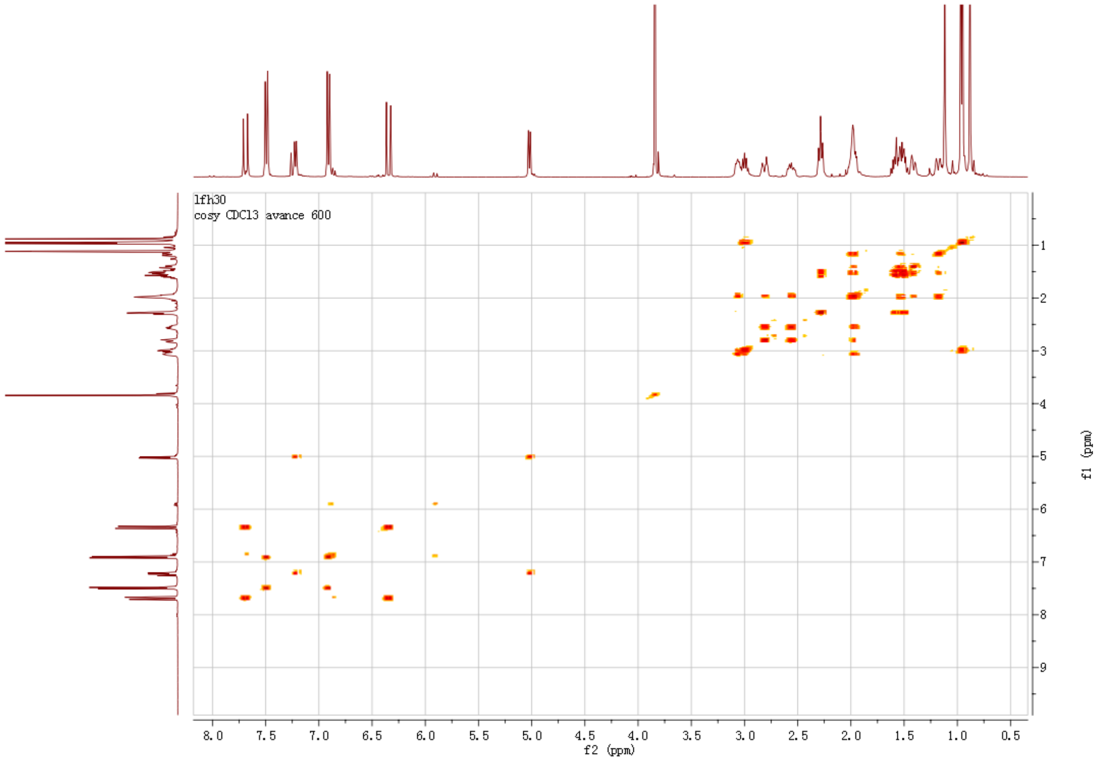

Figure S1.4. ^{1}H,^{1}H-COSY spectrum of compound **1** in CDCl3.

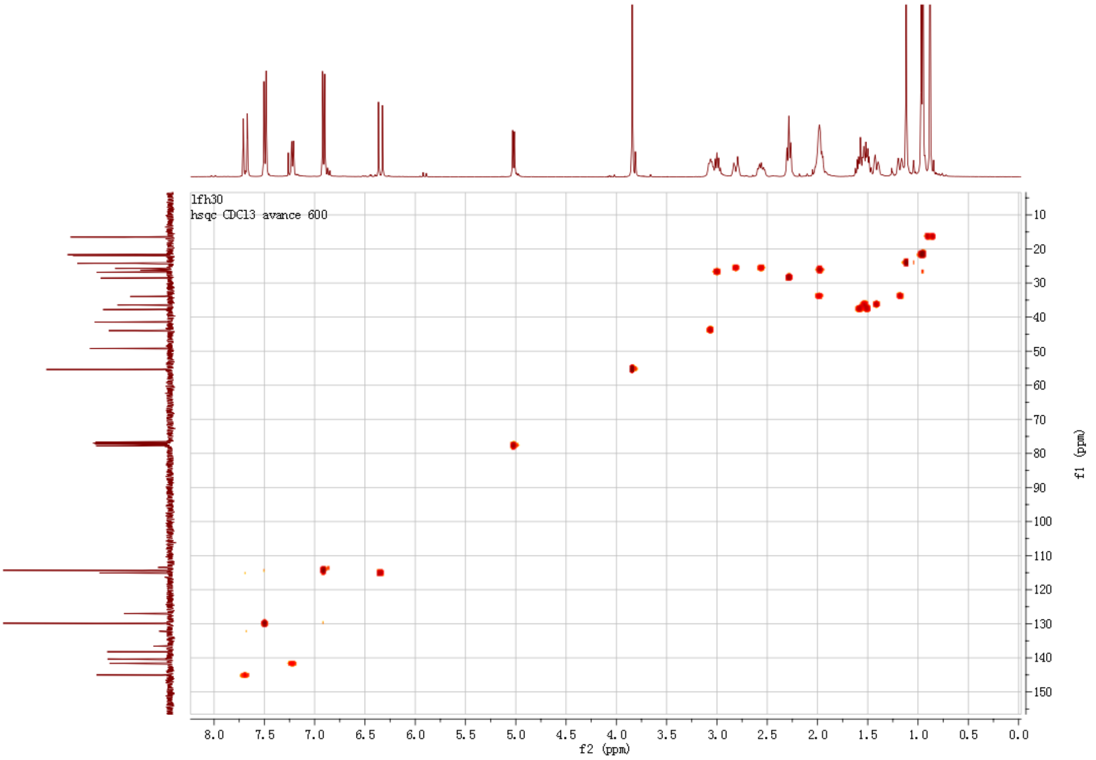

Figure S1.5. HSQC spectrum of compound **1** in CDCl3.

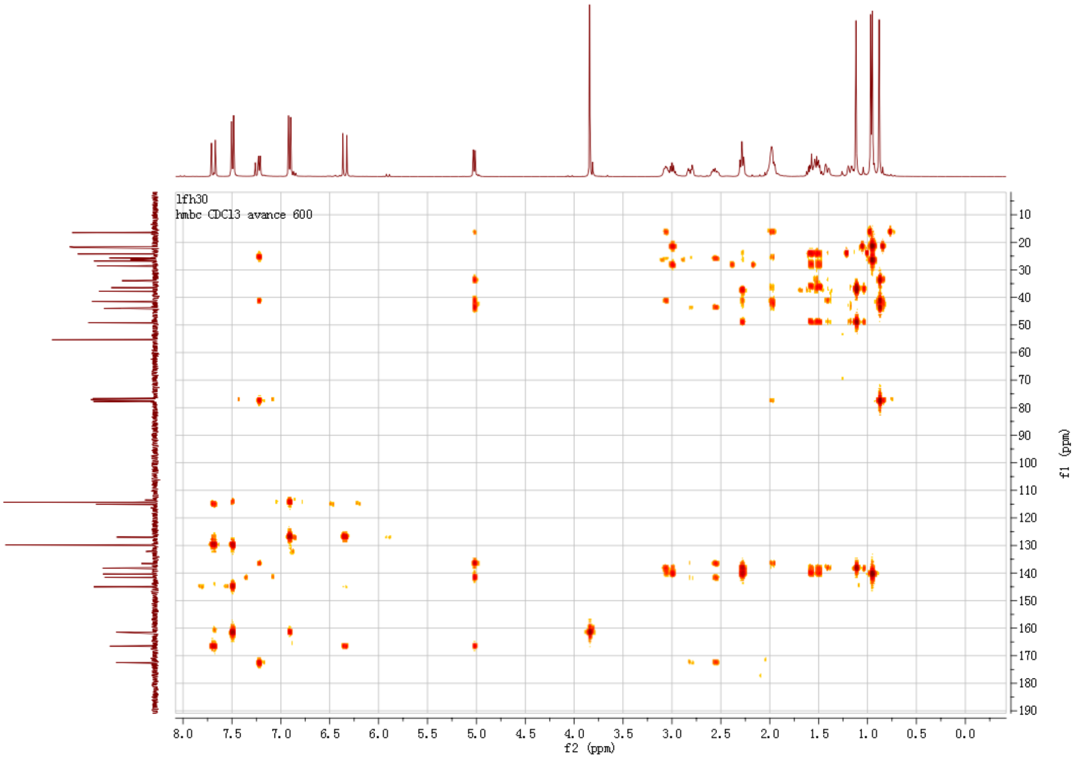

Figure S1.6. HMBC spectrum of compound **1** in CDCl3.

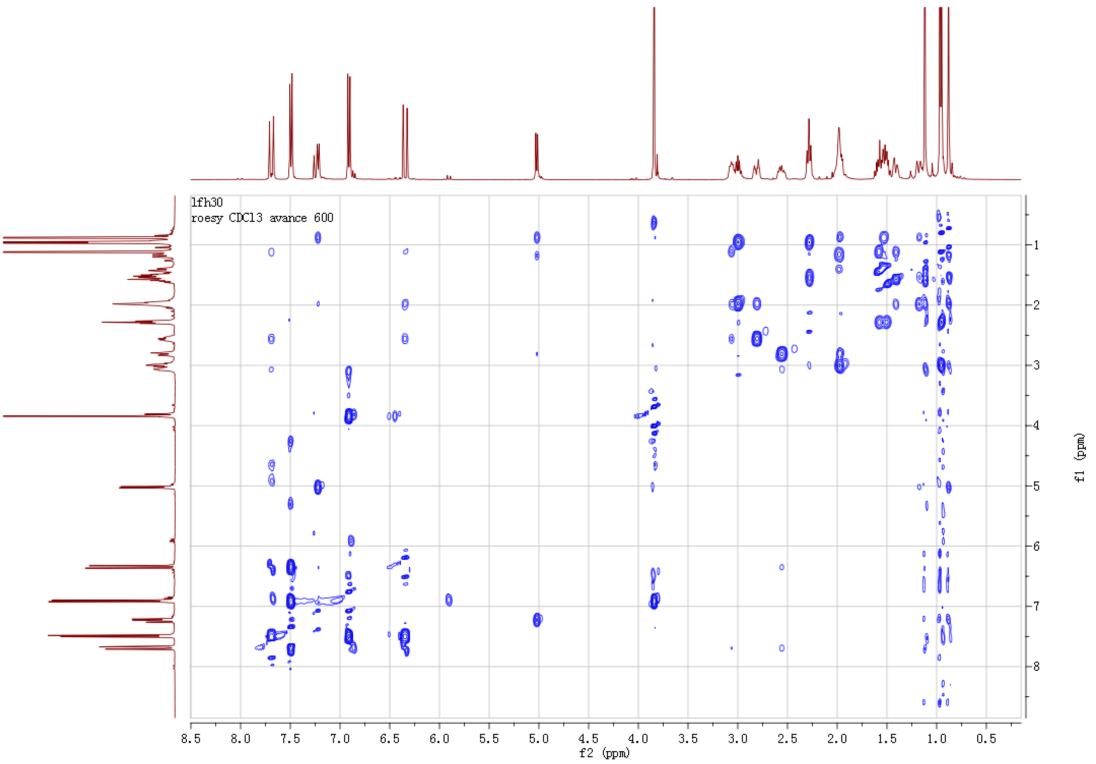

Figure S1.7. ROESY spectrum of compound **1** in CDCl3.

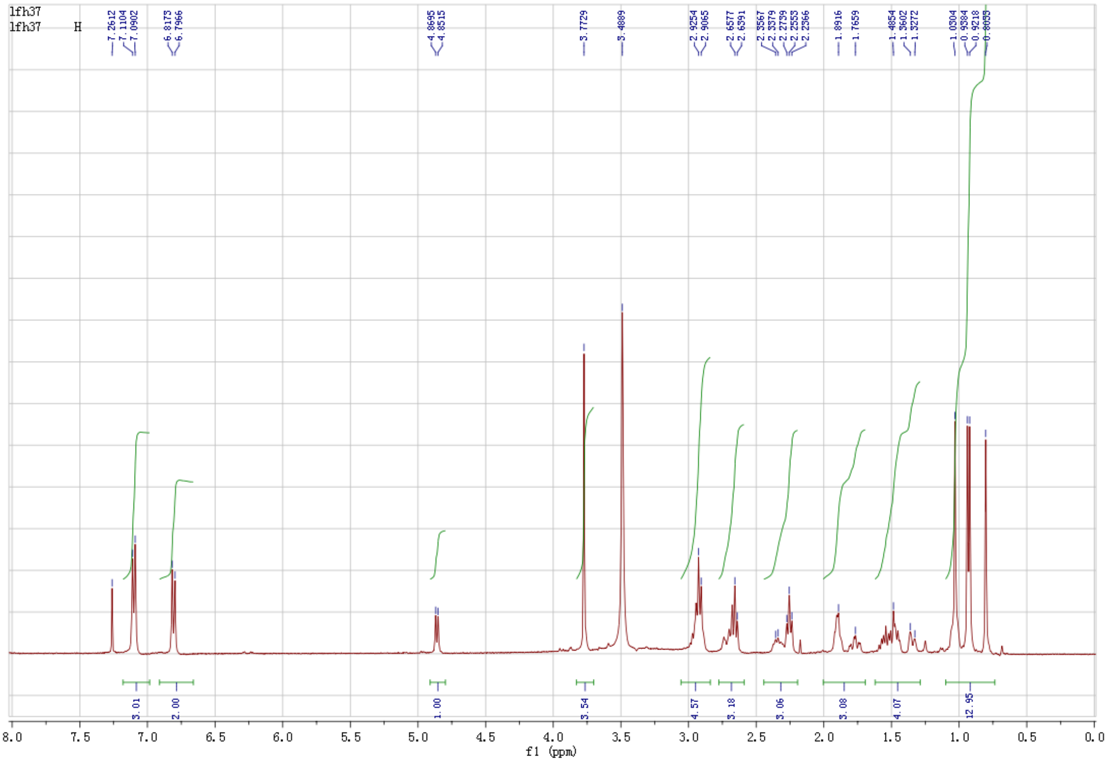

Figure S2.1. ^{1}H NMR spectrum of compound **2** in CDCl3.

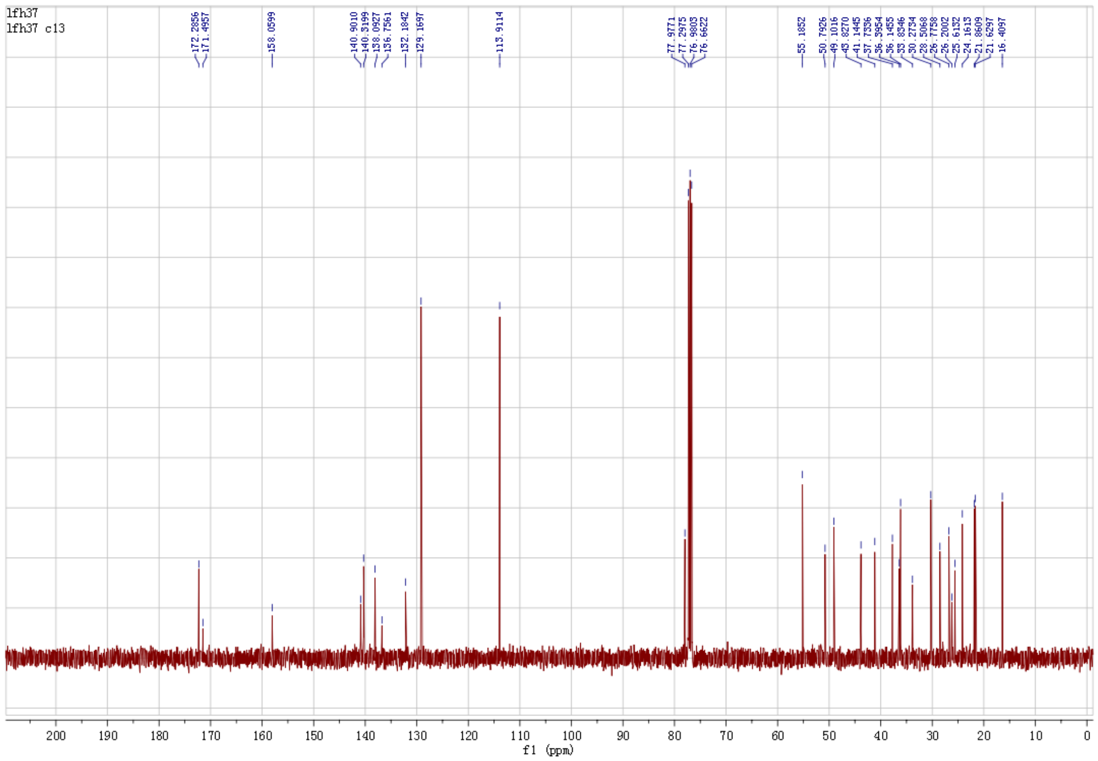

Figure S2.2. ^{13}C NMR spectrum of compound **2** in CDCl3.

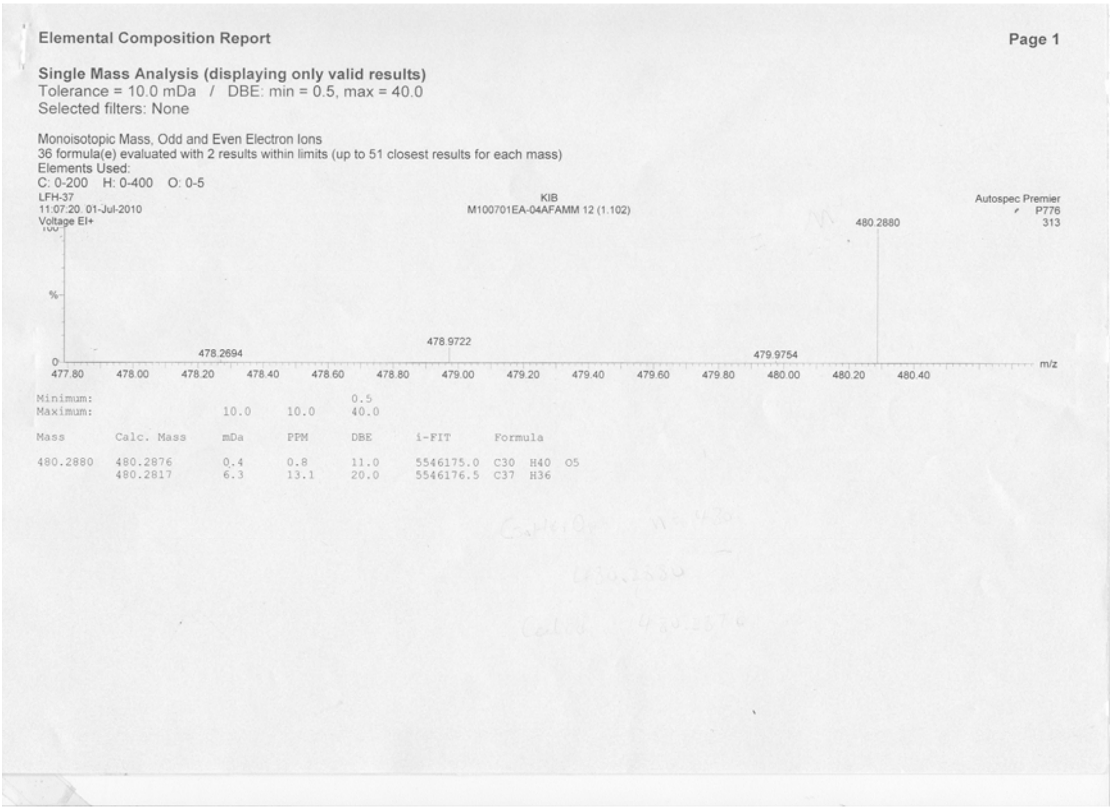

Figure S2.3. HR-EI-MS of compound **2**.

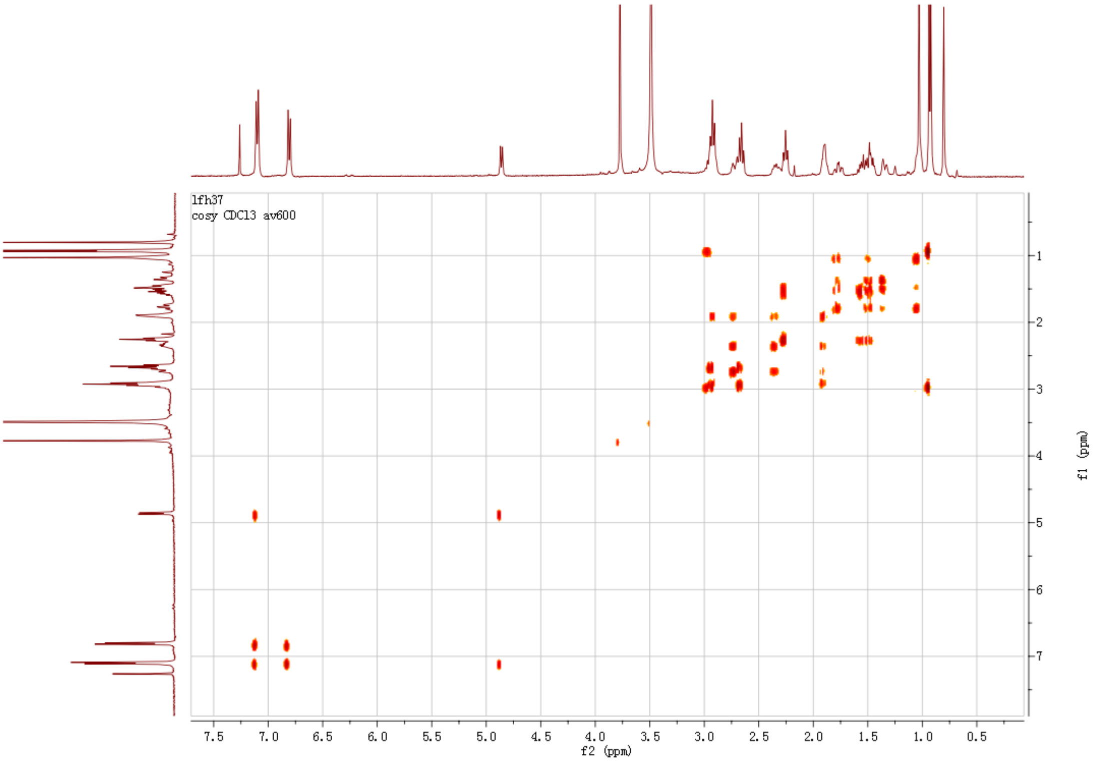

Figure S2.4. $^{1}H,^{1}H$-COSY spectrum of compound **2** in CDCl3.

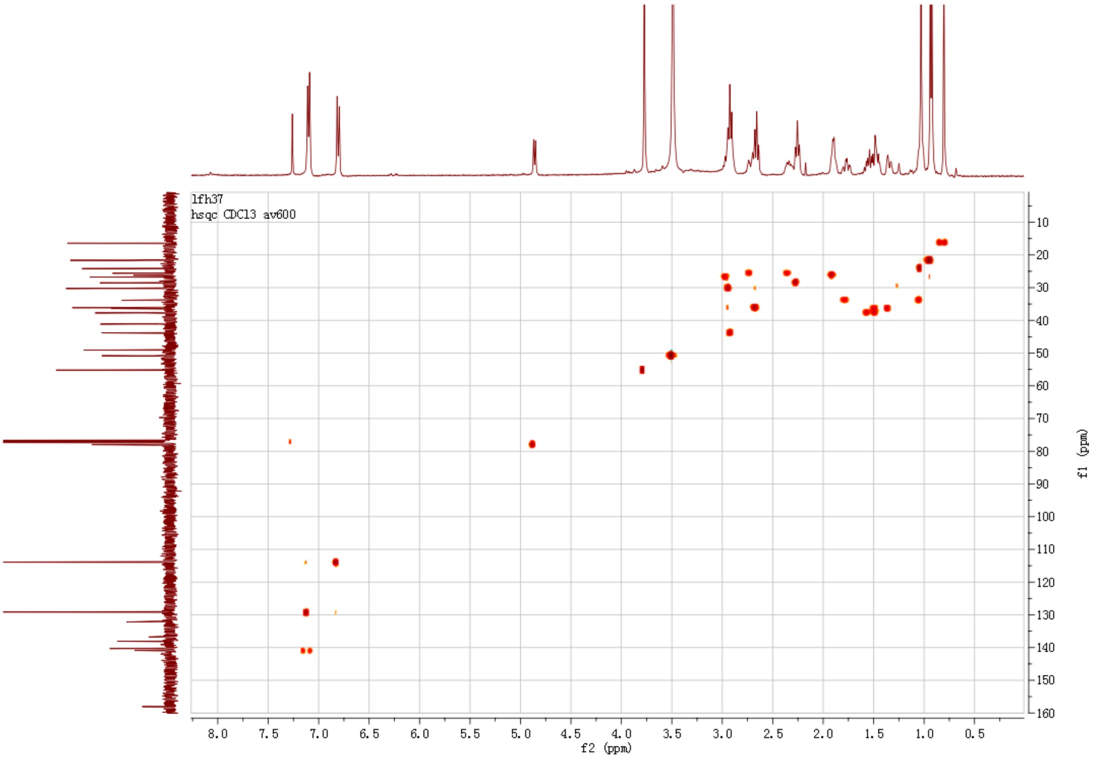

Figure S2.5. HSQC spectrum of compound **2** in CDCl3.

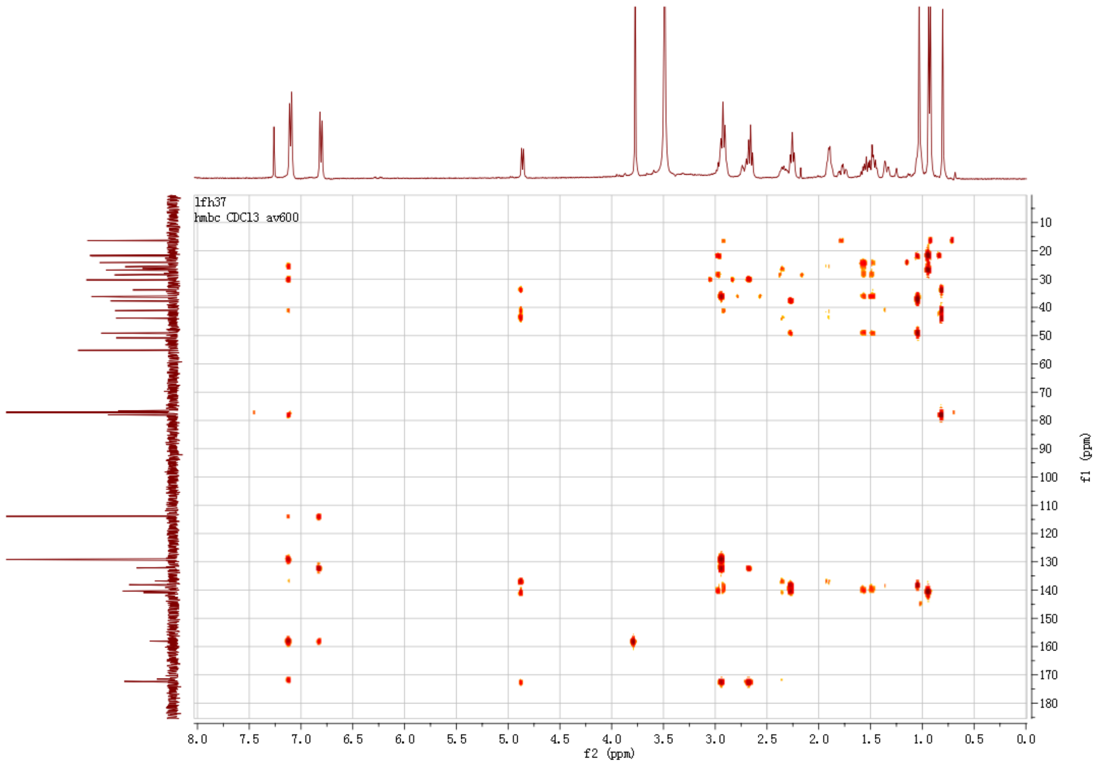

Figure S2.6. HMBC spectrum of compound **2** in CDCl3.

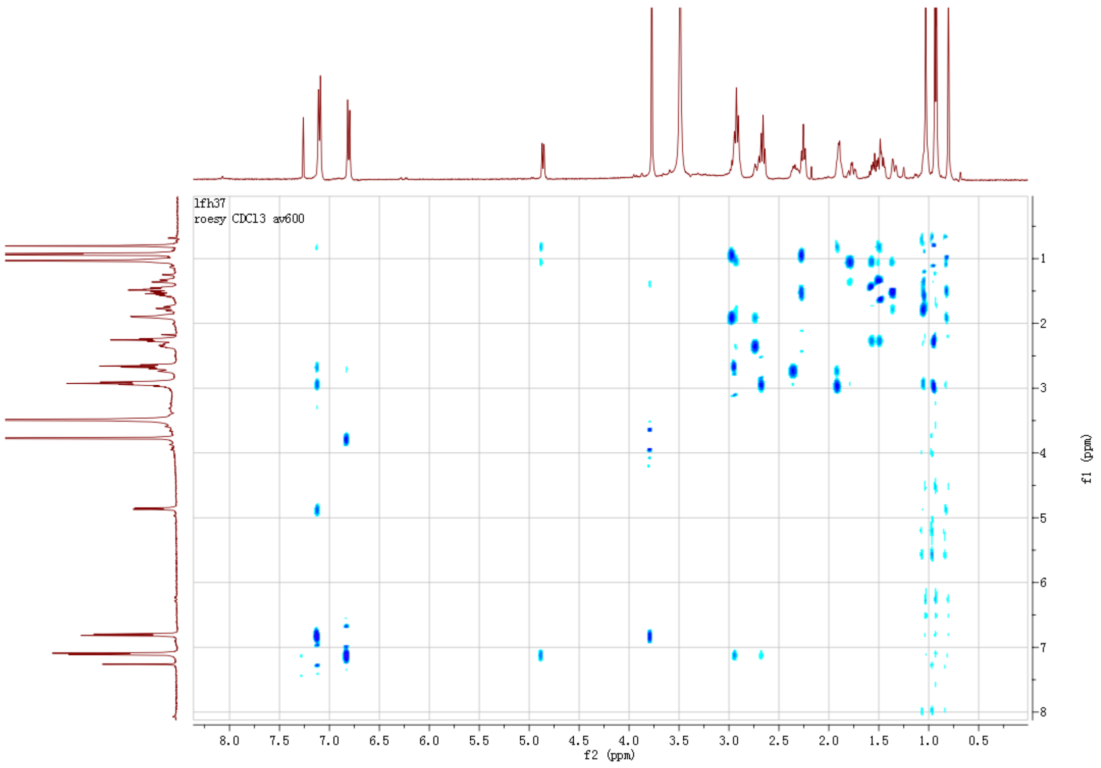

Figure S2.7. ROESY spectrum of compound **2** in CDCl3.

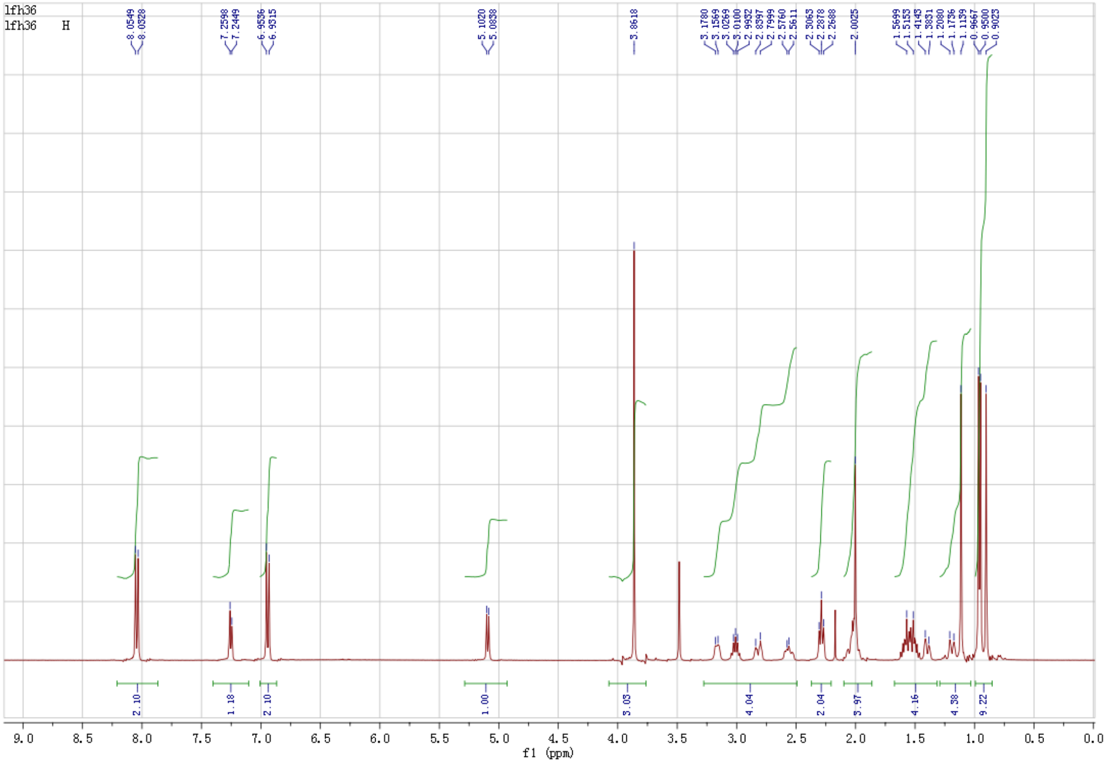

Figure S3.1. ^{1}H NMR spectrum of compound **3** in CDCl3.

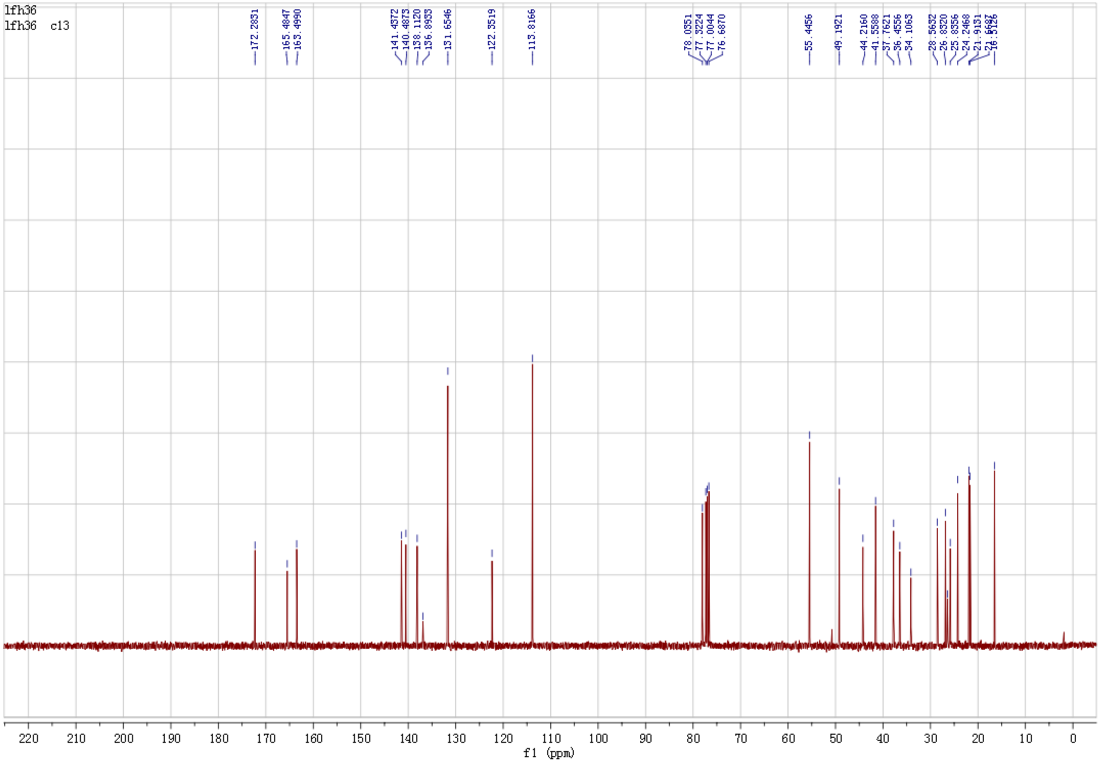

Figure S3.2. ^{13}C NMR spectrum of compound **3** in CDCl3.

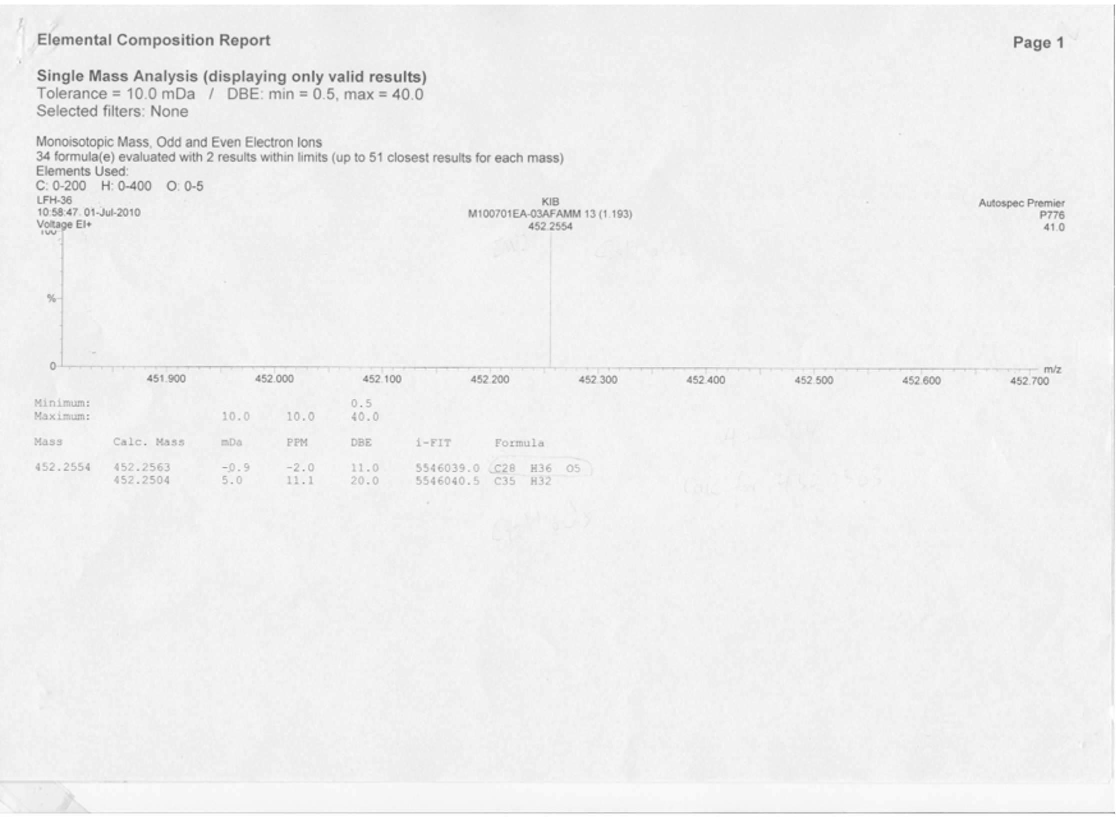
Elemental Composition Report

Page 1

Single Mass Analysis (displaying only valid results)
Tolerance = 10.0 mDa / DBE: min = 0.5, max = 40.0
Selected filters: None

Monoisotopic Mass, Odd and Even Electron Ions
34 formula(e) evaluated with 2 results within limits (up to 51 closest results for each mass)
Elements Used:
C: 0-200 H: 0-400 O: 0-5

LFH-36
10:58:47 01-Jul-2010
Voltage EI+

KIB
M100701EA-03AFAMM 13 (1.193)
452.2554

Autospec Premier
P776
41.0

Minimum:				0.5		
Maximum:		10.0	10.0	40.0		
Mass	Calc. Mass	mDa	PPM	DBE	i-FIT	Formula
452.2554	452.2563	-0.9	-2.0	11.0	5546039.0	C28 H36 O5
	452.2504	5.0	11.1	20.0	5546040.5	C35 H32

Figure S3.3. HR-EI-MS of compound **3**.

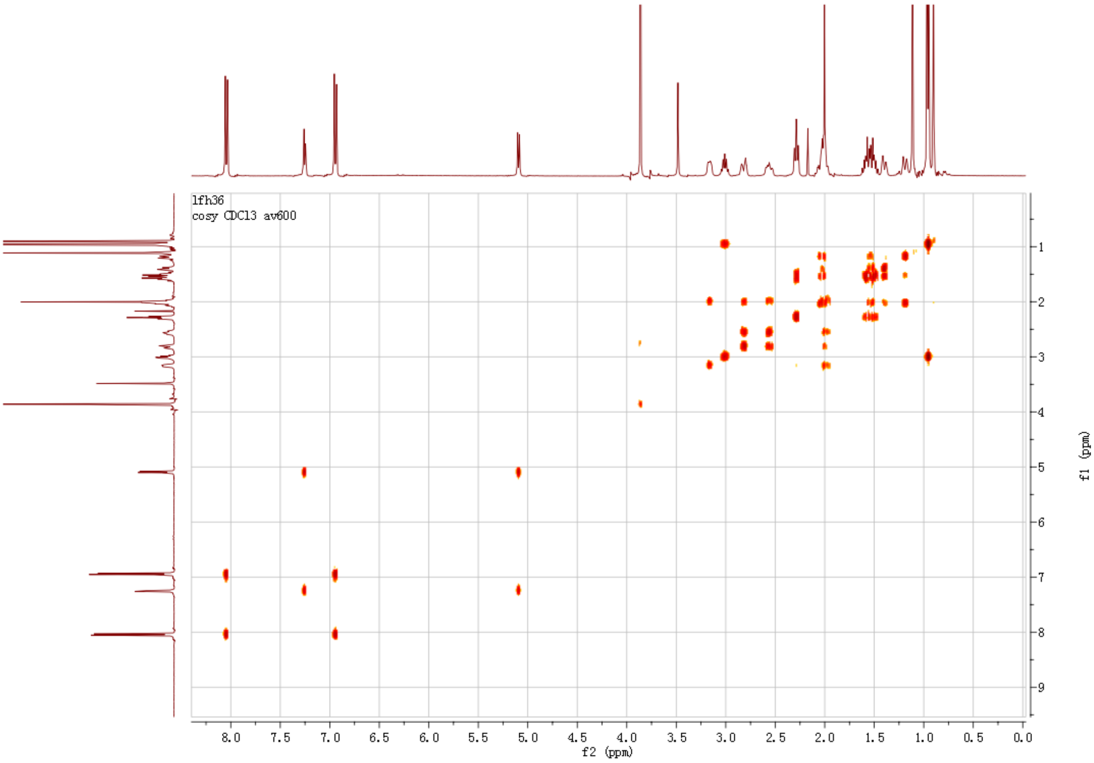

Figure S3.4. $^{1}H,^{1}H$-COSY spectrum of compound **3** in CDCl3.

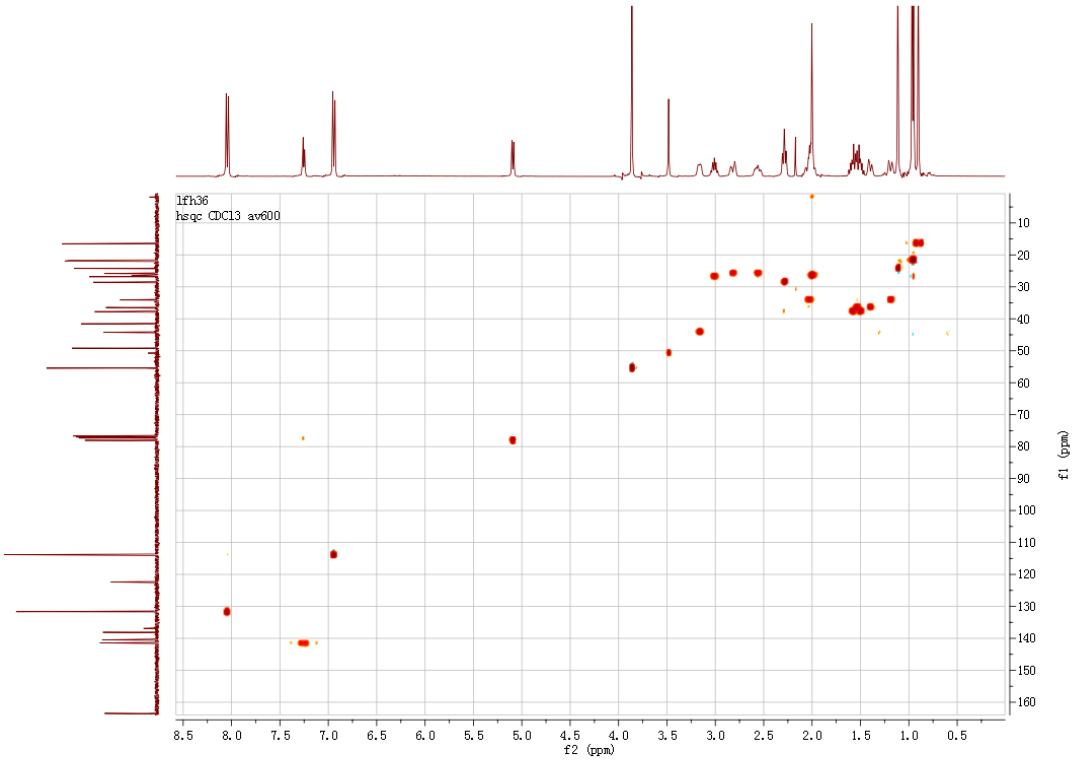

Figure S3.5. HSQC spectrum of compound **3** in CDCl3.

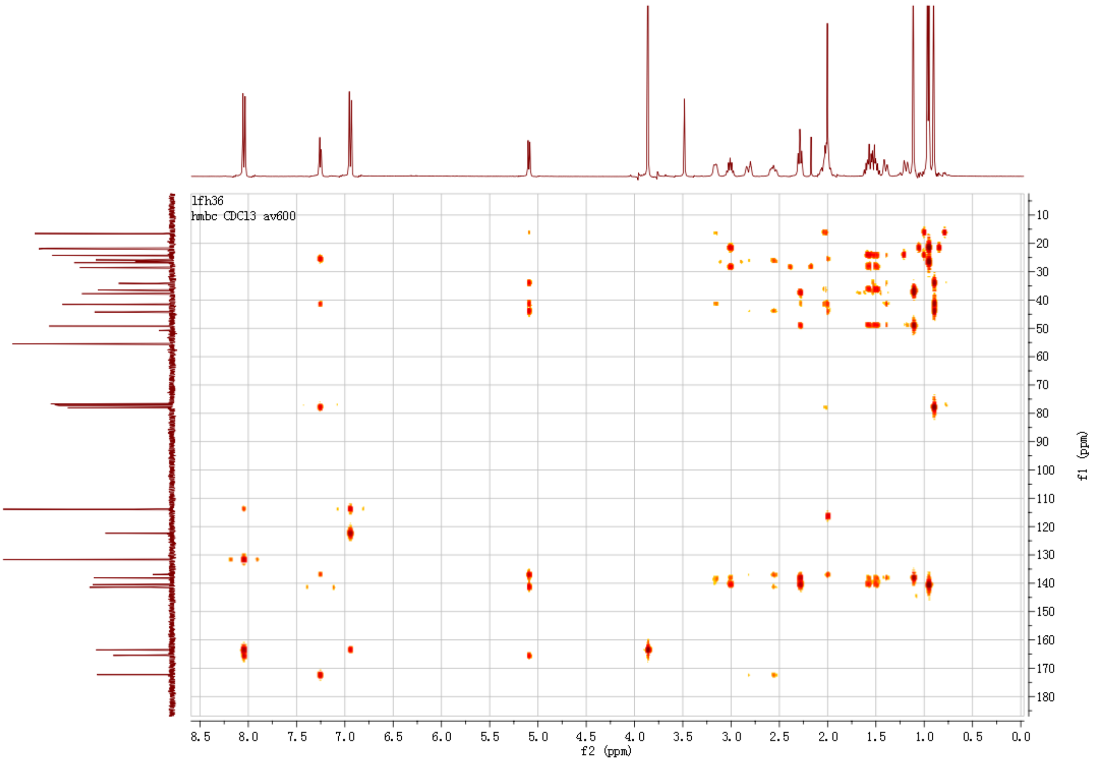

Figure S3.6. HMBC spectrum of compound **3** in CDCl3.

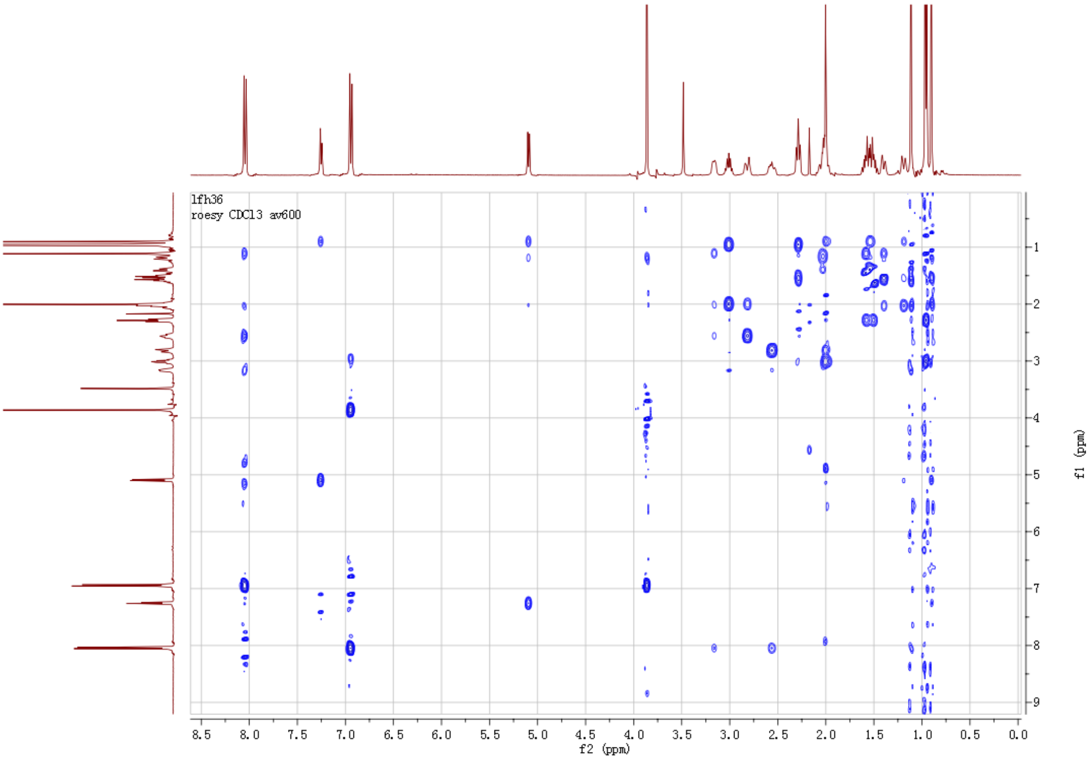

Figure S3.7. ROESY spectrum of compound **3** in CDCl3.

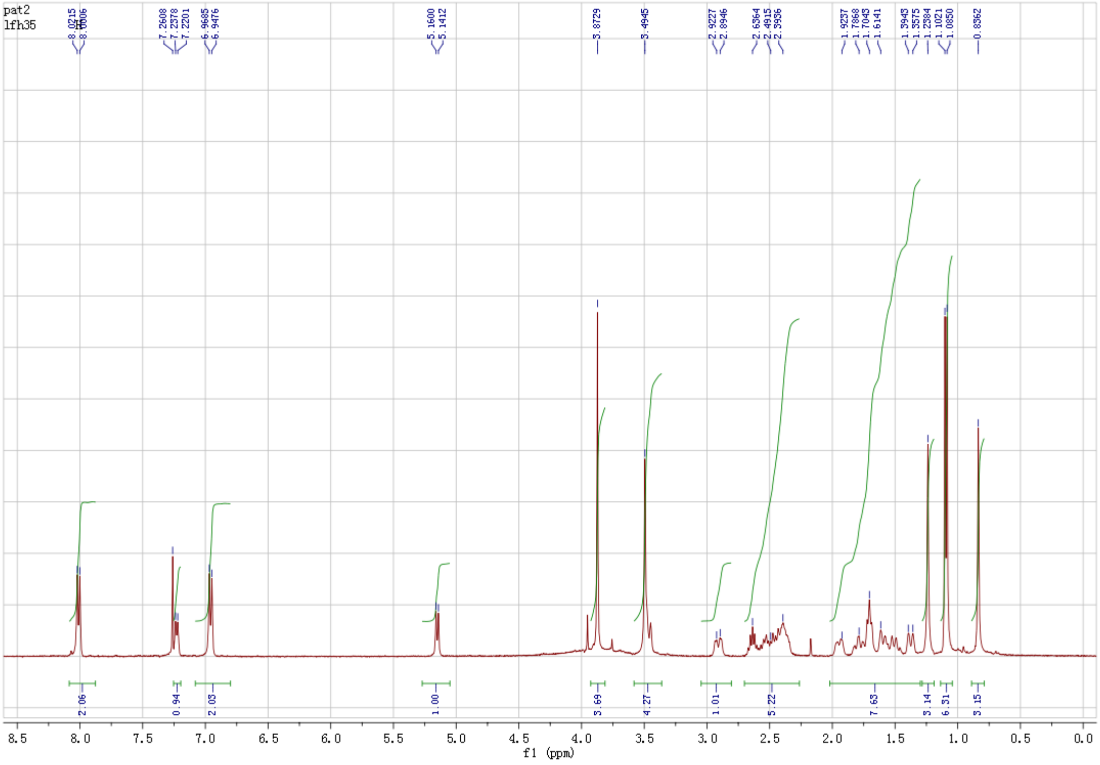

Figure S4.1. ^{1}H NMR spectrum of compound **4** in CDCl3.

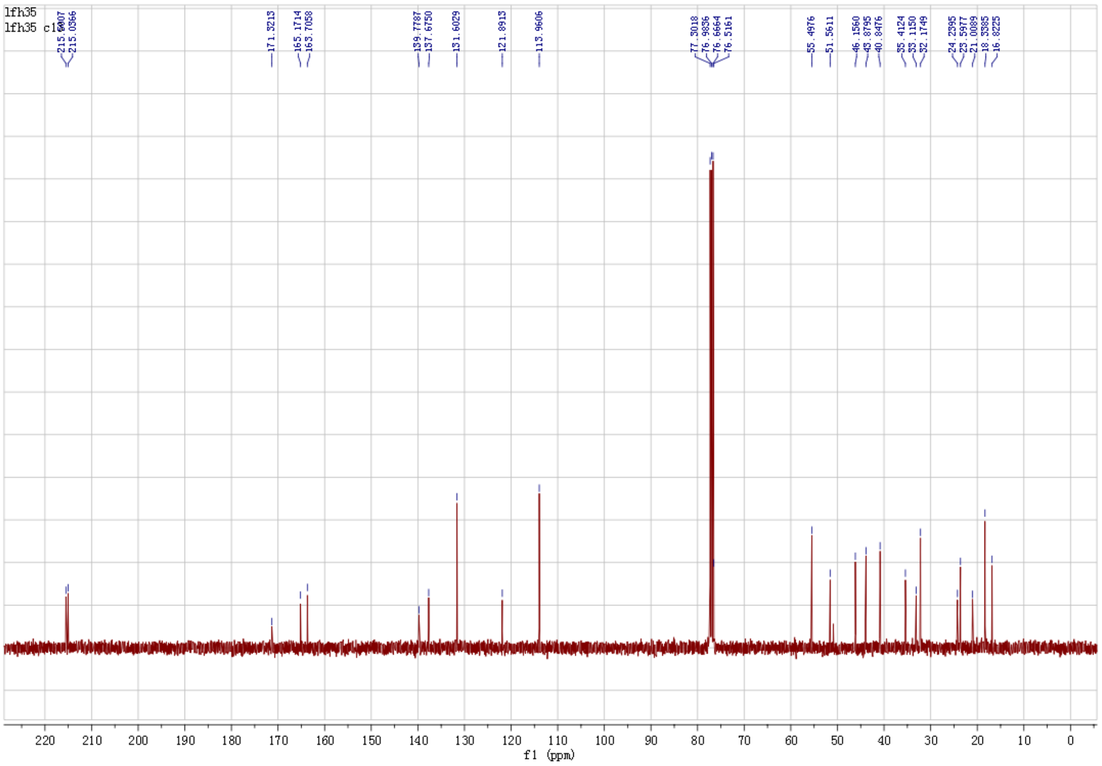

Figure S4.2. ^{13}C NMR spectrum of compound **4** in CDCl3.

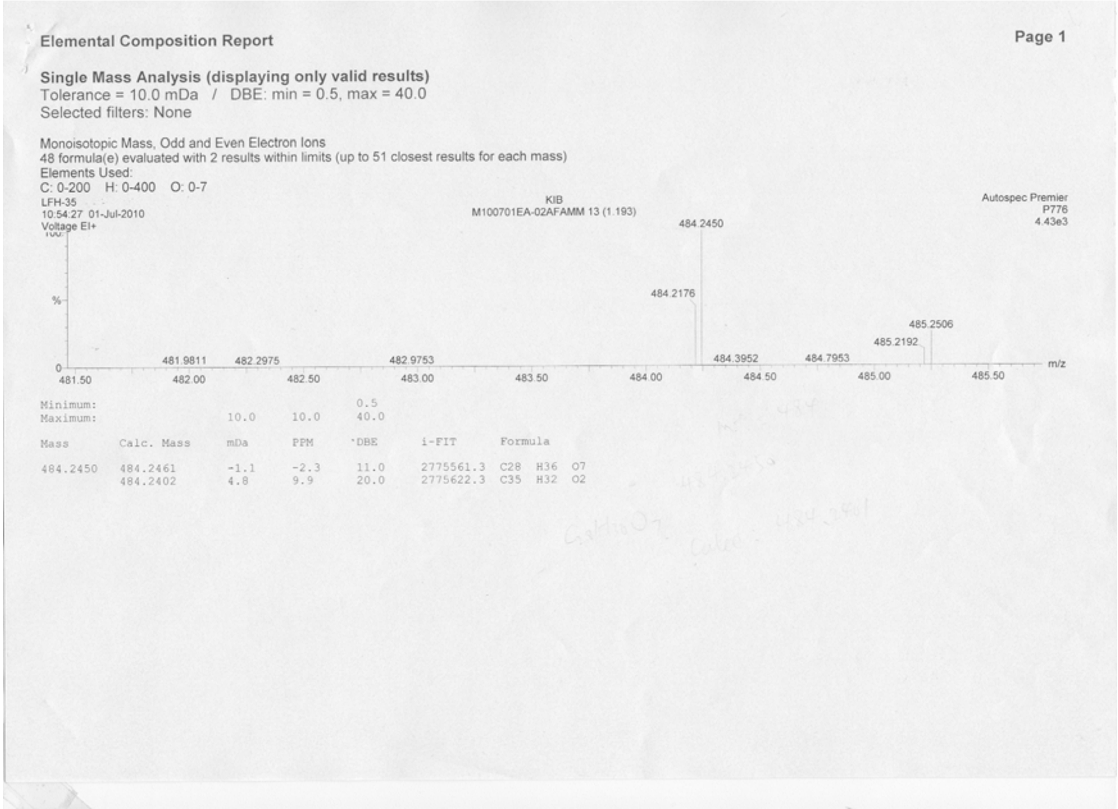

Elemental Composition Report

Page 1

Single Mass Analysis (displaying only valid results)
Tolerance = 10.0 mDa / DBE: min = 0.5, max = 40.0
Selected filters: None

Monoisotopic Mass, Odd and Even Electron Ions
48 formula(e) evaluated with 2 results within limits (up to 51 closest results for each mass)
Elements Used:
C: 0-200 H: 0-400 O: 0-7

Minimum:				0.5		
Maximum:		10.0	10.0	40.0		
Mass	Calc. Mass	mDa	PPM	DBE	i-FIT	Formula
484.2450	484.2461	-1.1	-2.3	11.0	2775561.3	C28 H36 O7
	484.2402	4.8	9.9	20.0	2775622.3	C35 H32 O2

Figure S4.3. HR-EI-MS of compound **4**.

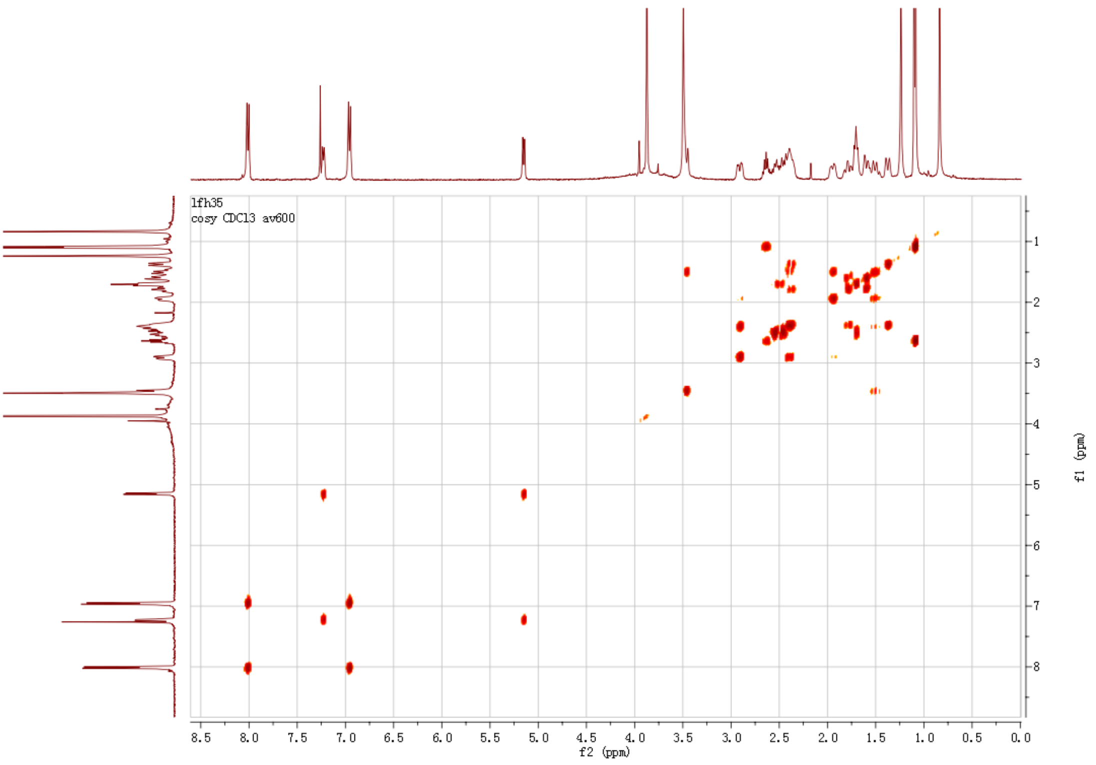

Figure S4.4. ^{1}H,^{1}H-COSY spectrum of compound **4** in CDCl3.

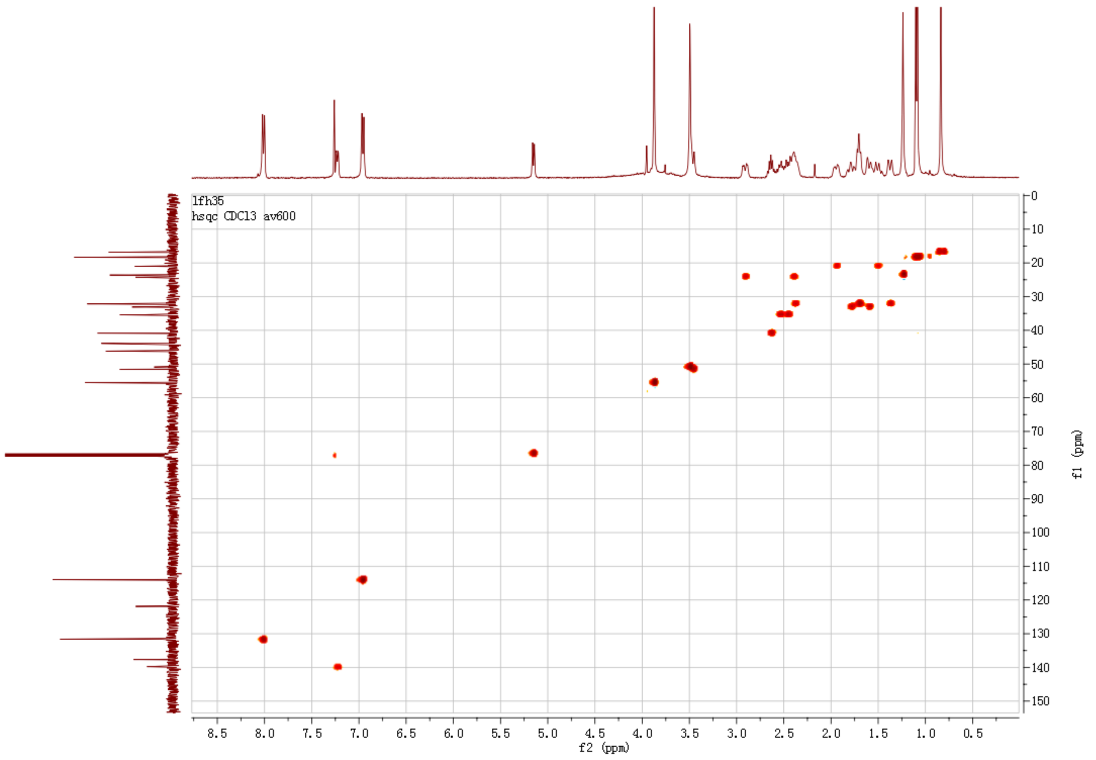

Figure S4.5. HSQC spectrum of compound **4** in CDCl3.

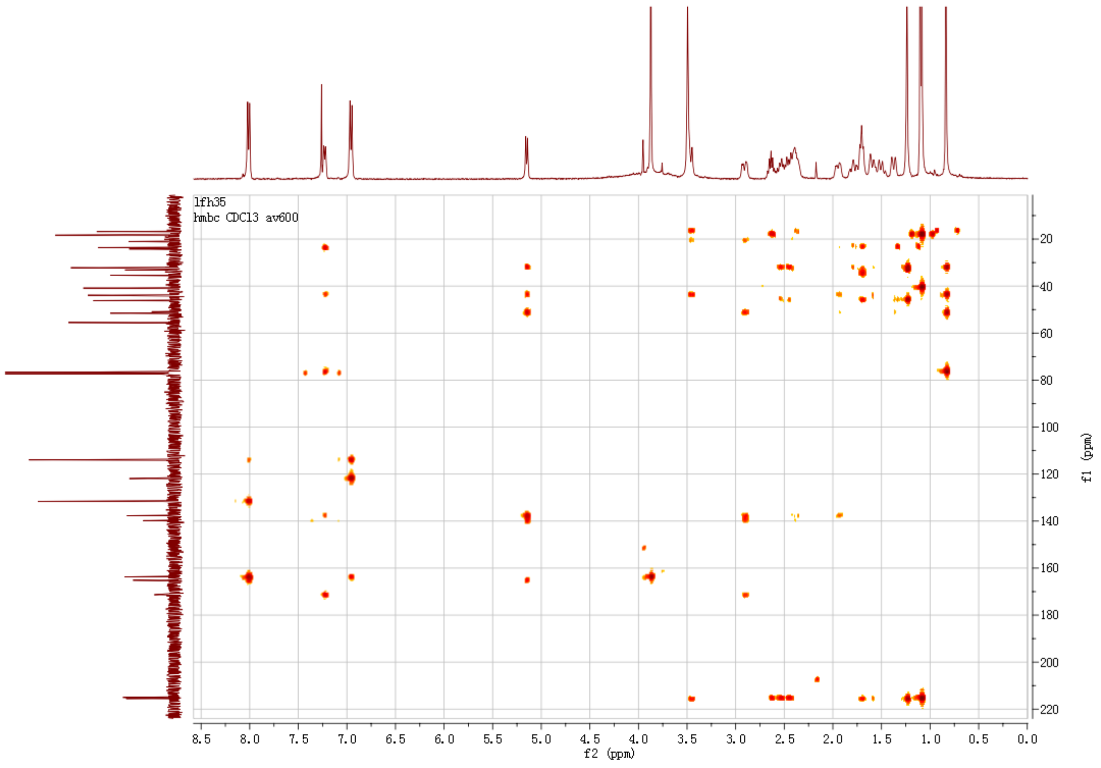

Figure S4.6. HMBC spectrum of compound **4** in CDCl3.

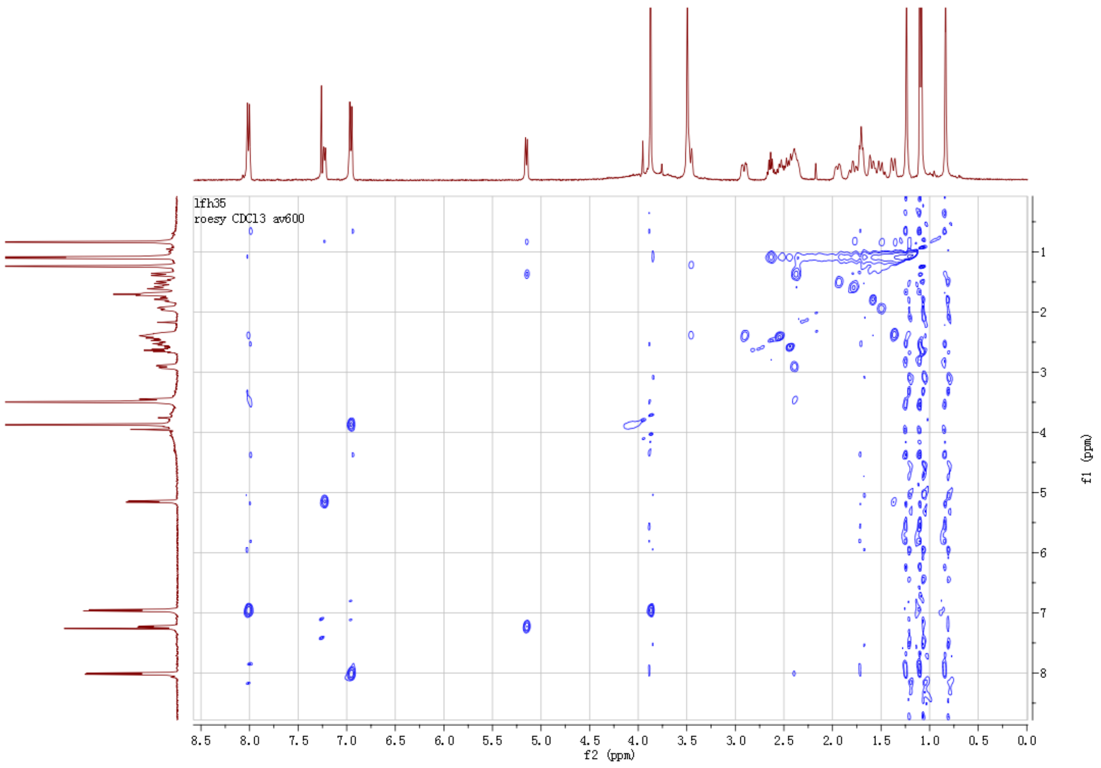

Figure S4.7. ROESY spectrum of compound **4** in CDCl3.
